# Mitogenomic diversity and phylogeny analysis of yak (*Bos grunniens*)

**DOI:** 10.1186/s12864-021-07650-x

**Published:** 2021-05-05

**Authors:** Xingdong Wang, Jie Pei, Pengjia Bao, Mengli Cao, Shaoke Guo, Rende Song, Weiru Song, Chunnian Liang, Ping Yan, Xian Guo

**Affiliations:** 1 Key Laboratory of Yak Breeding Engineering of Gansu Province, Lanzhou Institute of Husbandry and Pharmaceutical Sciences, Chinese Academy of Agricultural Sciences, Lanzhou, 730050 China; 2 Life Science and Engineering College, Northwest University for Nationalities, Lanzhou, 730030 China; 3 Animal Disease Prevention and Control Center of Yushu Tibetan Autonomous Prefecture, Yushu, 815000 China

**Keywords:** Genetic diversity, mtDNA, Phylogenetic relationship, Yak

## Abstract

**Background and aim:**

Mitochondrial genome has aseries of characteristics such as simple structure, no recombination, maternalinheritance, stable structure, fast evolution rate, and high copy number. Moreover, it is easy to be sequenced,contains high-resolution phylogenetic information, and exists in a wide rangeof taxa. Therefore, it is widely used in the study of biological phylogeny. Atpresent, phylogenetic studies focus mainly on D-loop region, cytochrome b gene,and protein-coding sequence. Phylogenetic studies using the mitochondrialcomplete sequence are rarely reported in yak. Therefore, the present studyaimed to construct phylogenetic tree using yak mitochondrial complete sequenceand compare the subsequent results with previous findings obtained usingpartial sequences.

**Results:**

Complete mitochondrial sequences of five yakpopulations from Qinghai and Xinjiang were obtained. The mitotype diversity ofthe five populations was Xueduo yak (0.992 ± 0.015), Pamir yak (0.990 ± 0.014),Yushu yak (0.963 ± 0.033), Qilian yak (0.948 ± 0.036), and Huanhu yak (0.905 ±0.048), which showed a higher mitotype diversity compared with other breeds fromthe previous reports, including Jiulong yak, Maiwa yak, Zhongdian yak, andTianzhu yak. A total of 78 mitotypes were obtained from 111 individuals. Amongthese, Yushu yak, Huanhu yak, Xueduo yak, and Qilian yak all shared mitotypes,but the Pamir yak did not share mitotypes with these four populations.Phylogenetic analysis showed that yak populations were separable into threedistinct branches. The analysis identified a new phylogenetic branch containingboth wild and domestic yaks. The 155 mitotypes found in 206 individuals weredivided into 3 haplogroups by mitotype clustering. Thehaplogroup was not associated with the geographical distribution of yaks. Theyaks in the same population or the same ecological environment were distributedin different haplogroups. Among the threehaplogroups, haplogroup A and haplogroup B showed a star-shaped distribution ofmitotypes. The central mitotypes were widely distributed and had a highfrequency.

**Conclusions:**

Thegenetic diversity of yaks in Qinghai was high. Both domestic and wild yaks clusteredinto three branches.

**Supplementary Information:**

The online version contains supplementary material available at 10.1186/s12864-021-07650-x.

## Background

Yak (*Bos grunniens*) is a large animal with a compact body, an absence of functioning sweat glands, and a relatively small skin surface area per unit of body weight [1]. These characteristics make yaks well adapt to low-temperature hypoxic environments where they are endemic, such as the Qinghai−Tibetan Plateau (QTP) and adjacent high-altitude regions [2]. China has more than 16 million yaks [3], accounting for over 95 % of the global yak population [4]. Yaks are multipurpose, high-altitude bovid species [5], providing an essential source of economically valuable products, such as milk, meat, fur, and fuel, for local herders [6]. In addition, yaks provide locals with a means of transportation [7]. Thus, yaks play an important socioeconomic role in the regions where they are endemic, being vital in maintaining pasture ecosystems and agricultural biodiversity in plateau areas [8]. Hence, yaks are referred to as “all-around animals” [2]. The statistical results in recent years showed that the body weight and meat yield of yaks have decreased significantly compared with yaks 10 years ago; yaks are facing serious degradation and hence it is urgent to improve yak breeds. Yaks may have been domesticated from wild yaks by ancient Qiang people in northwest China during the early Holocene period [7]. They are the only large animals live in the low-temperature hypoxic environment to which their wild ancestors adopt [9]. Qinghai province is located in the northern QTP, abundant of yak genetic resources. Archeological analyses and assessments of mitochondrial DNA (mtDNA) sequences suggest that Qinghai province may be the origin area of yak domestication [10]. However, there has laid a foundation for further research is warranted to better clarify the maternal origins, phylogenetic structure, and diversity of domestic yak populations by studying the yak mitochondrial genome.

 Genetic diversity is a central facet of biological diversity [11], and variations in DNA sequences are the primary drivers of such diversity. MtDNA is a self-replicating, maternally inherited circular DNA molecule that undergoes rapid evolution and no recombination, and contains high frequencies of polymorphic variants [12]. As the entire mitochondrial genome is inherited as a single unit and mutations accumulate therein [13], mtDNA is considered an ideal tool for performing animal studies, evolution, classification, and population genetic diversity [14]. Previous studies analyzed mtDNA to explore bovine genetic diversity [15]. A separate analysis of mtDNA samples from domestic yaks revealed that all domestic yaks originated from a common wild yak ancestor [15], enabling researchers to tentatively identify two domestic yak migration routes [14]: One originates from the eastern part of the QTP through the Himalayas and the Kunlun Mountains to the Pamir region, and the other originates from the eastern part of the QTP through the South Gobi and the Altai Mountains to Mongolia and Russia. Diversity levels of the domestic yak population are the highest in the QTP region [14]. Most mtDNA studies conducted to date have focused explicitly on the control D-loop region of the mitochondrial genome, due to its relative less genetic information, it virtually impossible to clearly distinguish certain important branches in livestock [16, 17]. Recently, researchers have discussed the importance of performing complete mtDNA sequencing because such analyses can yield a detailed genetic map when conducted with a sufficiently large sample size [18].

Yaks from Qinghai province were selected as the research object and Pamir yaks from Xinjiang province were considered as the comparison group in this study. The complete mitochondrial sequences of Yushu, Qilian, Huanhu, Xueduo, and Pamir populations were obtained by sequencing, and the genetic diversity and interspecific genetic distance of the five populations were analyzed. The phylogenetic relationship of yaks was analyzed using the sequencing data and the existing mitochondrial sequences data in GenBank. This study evaluated the genetic diversity of the yak’s complete mitochondrial DNA at the molecular level and laid a foundation for effectively protecting and using bovine resources.

## Results

### Analysis of polymorphic sites of mtDNA

**Fig. 1:**
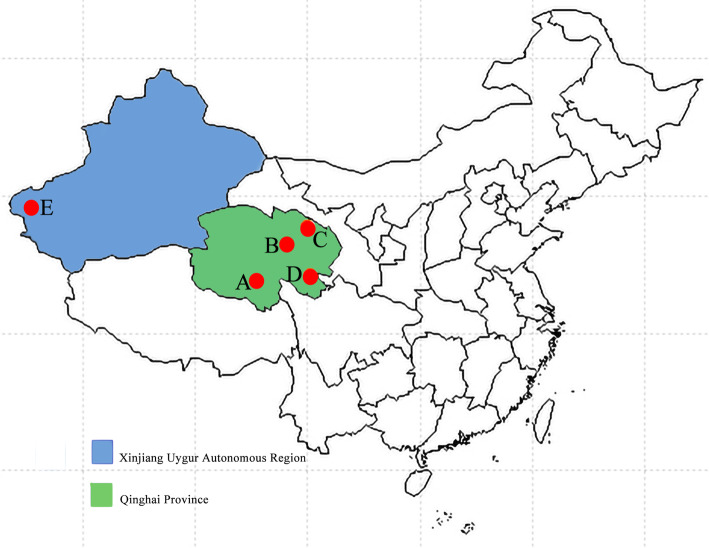
Collectionsite of yak blood samples: A, B, C, D, and E. A was the collection area of Yushu yak, B was the collection area of Huanhu yak, C was the collection area of Qilian yak, D was the collection area of Xueduo yak, and E was the collection area of Pamir yak

 This study assessed genetic variations in 111 complete mtDNA sequences (16,321–16,325 bp) of four yak populations from Qinghai province and one yak population from Xinjiang province to evaluate the genetic diversity, and phylogeny of these populations. Reference to the geographic location of yak blood sample collection is shown in Fig. 1. A total of 114 variable sites were found in Huanhu yaks (1 singleton variable site and 113 parsimony informative sites), 105 in Pamir yaks (1 singleton variable site and 104 parsimony informative sites), 105 in Qilian yaks (all were parsimony informative sites), 122 in Xueduo yaks (all were parsimony informative sites), and 115 in Yushu yaks (all were parsimony informative sites). Through these sequence analyses across all 5 populations, 150 variable sites were found, including 2 singleton variable sites and 148 parsimony informative sites.

### Mitotype analysis of mtDNA

A total of 78 mitotypes were defined for these 111 samples. Among 78 mitotypes, 6 were shared by different populations (accounting for 7.7 % of the total mitotypes) and 72 were specific mitotypes of specific populations (accounting for 92.3 % of the total mitotypes). The number and type of mitotypes were different among different populations. Of these, mitotype H2 was the most common, being observed 13 times and represented among Huanhu, Qilian, Xueduo, and Yushu yaks. Of the 78 mitotypes observed in these 5 yak populations, only Pamir yaks in Xinjiang did not share mitotypes with other yak populations; the other 4 yak populations all shared mitotypes. More information on the mitotypes is available in Supplementary Table S1.

### Genetic diversity analysis of yak mtDNA

The genetic diversity analysis showed that the mitotype diversity of the five yak populations was 0.981 ± 0.007, and the nucleotide diversity was 0.00272 ± 0.00019. These data showed that the genetic diversity of five yak populations was high. The nucleotide diversity value of the Pamir yak population (0.00309 ± 0.00018) was higher than that of the other four populations. The mitotype diversity reached a maximum in the Xueduo yak population (0.992 ± 0.015) and a minimum in the Huanhu yak population (0.905 ± 0.048). The D-loop region was found to be the most variable mtDNA sequence in these yaks. Additional details pertaining to genetic diversity estimates, including the variable site number, mitotype number, and nucleotide diversity [Pi ± standard deviation (SD)], are shown in Table 1.

**Table 1:** Genetic structure and diversity of Qinghai and Pamir yaks

Population	N	Haplogroup			S	H	K	Hd ± SD	Pi ± SD
		A	B	C					
Huanhu	21	17	4	0	114	12	35.943	0.905 ± 0.048	0.00220 ± 0.00061
Pamir	25	13	11	1	105	22	50.447	0.990 ± 0.014	0.00309 ± 0.00018
Qilian	22	15	7	0	105	16	44.610	0.948 ± 0.036	0.00273 ± 0.00043
Xueduo	23	18	4	0	122	21	37.921	0.992 ± 0.015	0.00232 ± 0.00051
Yushu	20	13	5	1	115	16	45.005	0.963 ± 0.033	0.00276 ± 0.00047
Total	111	76	31	2	150	78	44.470	0.981 ± 0.007	0.00272 ± 0.00019

*H* Number of mitotypes, *Hd* haplotype diversity, *K* the average number of differences, *N* number of yaks, *Pi* nucleotide diversity, *S* number of variable sites, *SD* standard deviation

### Assessment of mtDNA sequence variations and genetic diversity

**Fig. 2:**
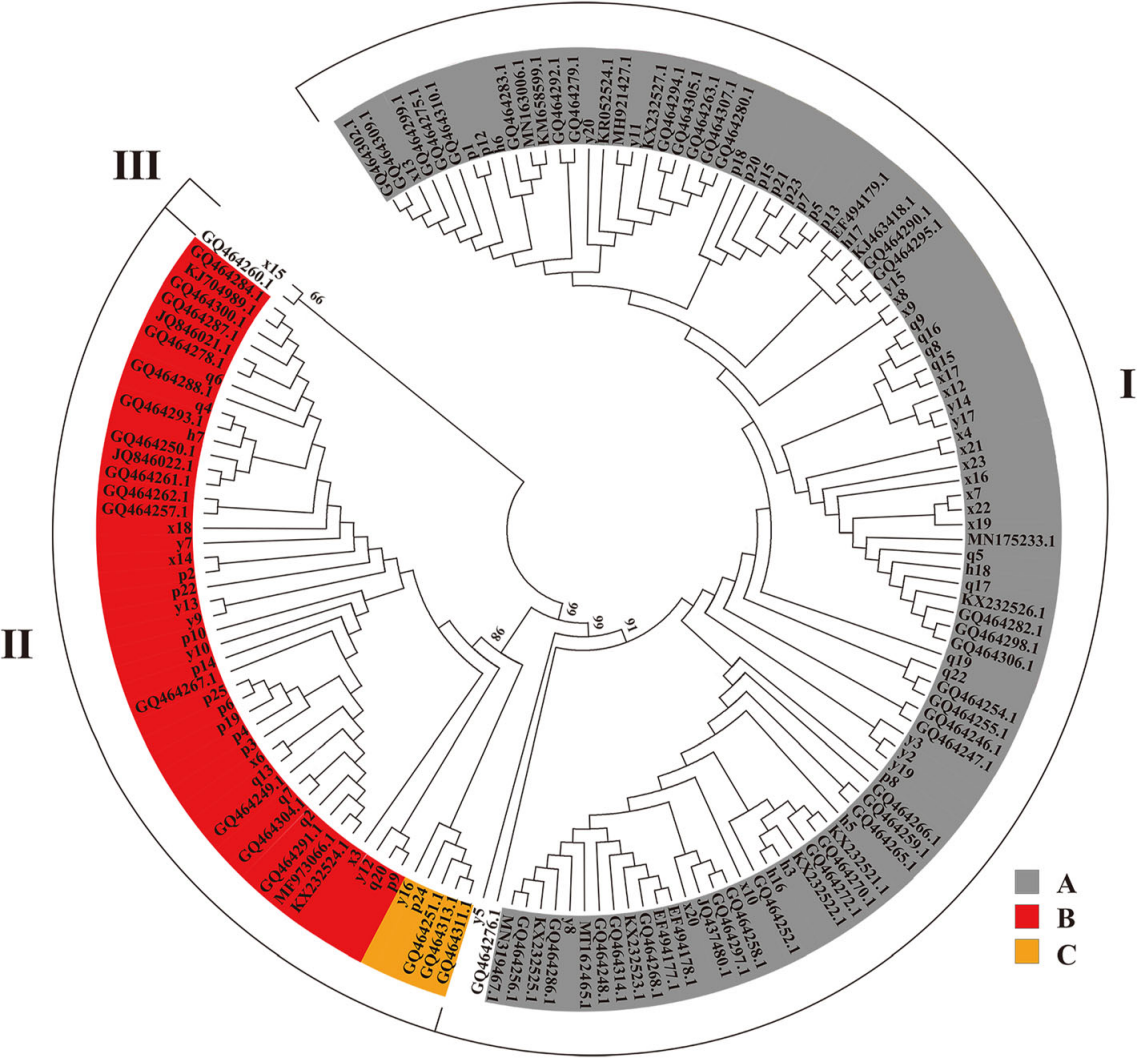
A total of 155 mitotypes of neighbor-joining trees in yaks. The reliability of the tree topology was assessed by 1000 bootstrap replicates. I represents branch 1, II represents branch 2, and III represents branch 3; A, B,and C represent the haplogroups. h represents the Huanhu yak, y represents the Yushu yak, x represents the Xueduo yak, q represents the Qilian yak, and p represents the Pamir yak. The remaining sequences were downloaded from National Center for Biotechnology Information and detailed in Supplementary Table S2.

Together with the 111 sequences obtained by sequencing, 95 complete mitochondrial genome sequences of yaks that lived in other provinces were downloaded from GenBank (Supplementary Table S2); 206 complete mitochondrial genome sequences contained 155 mitotype sequences (Supplementary Table S3). A neighbor-joining tree (Fig. 2) was next constructed based on these 155 mitotype sequences. This tree separated yaks into 3 branches, of which branch I was the largest (with 103 mitotypes), accounting for 66.45 % of the total mitotypes. Branch II contained 50 mitotypes, accounting for 32.26 % of the total mitotypes. Branch III was the smallest; only one domestic and one wild yak were included, accounting for 1.29 % of the total mitotypes. Phylogenetic analyses revealed that yak mtDNA was separable into three haplogroups (haplogroups A–C). Haplogroup A was the most frequent (accounting for two thirds of all mitotypes), followed by haplogroup B. At the same time, haplogroups A and B both included Yushu yak, Qilian yak, Xueduo yak, Huanhu yak, and Pamir yak from five populations. The haplogroup C had the lowest number and included only Yushu yak and Pamir yak individuals. The statistical analysis of the distribution of yaks in each province in the haplogroup revealed that haplogroup A included yaks from six provinces and haplogroup B included yaks from Qinghai, Sichuan, Gansu, and Xinjiang.

**Fig. 3:**
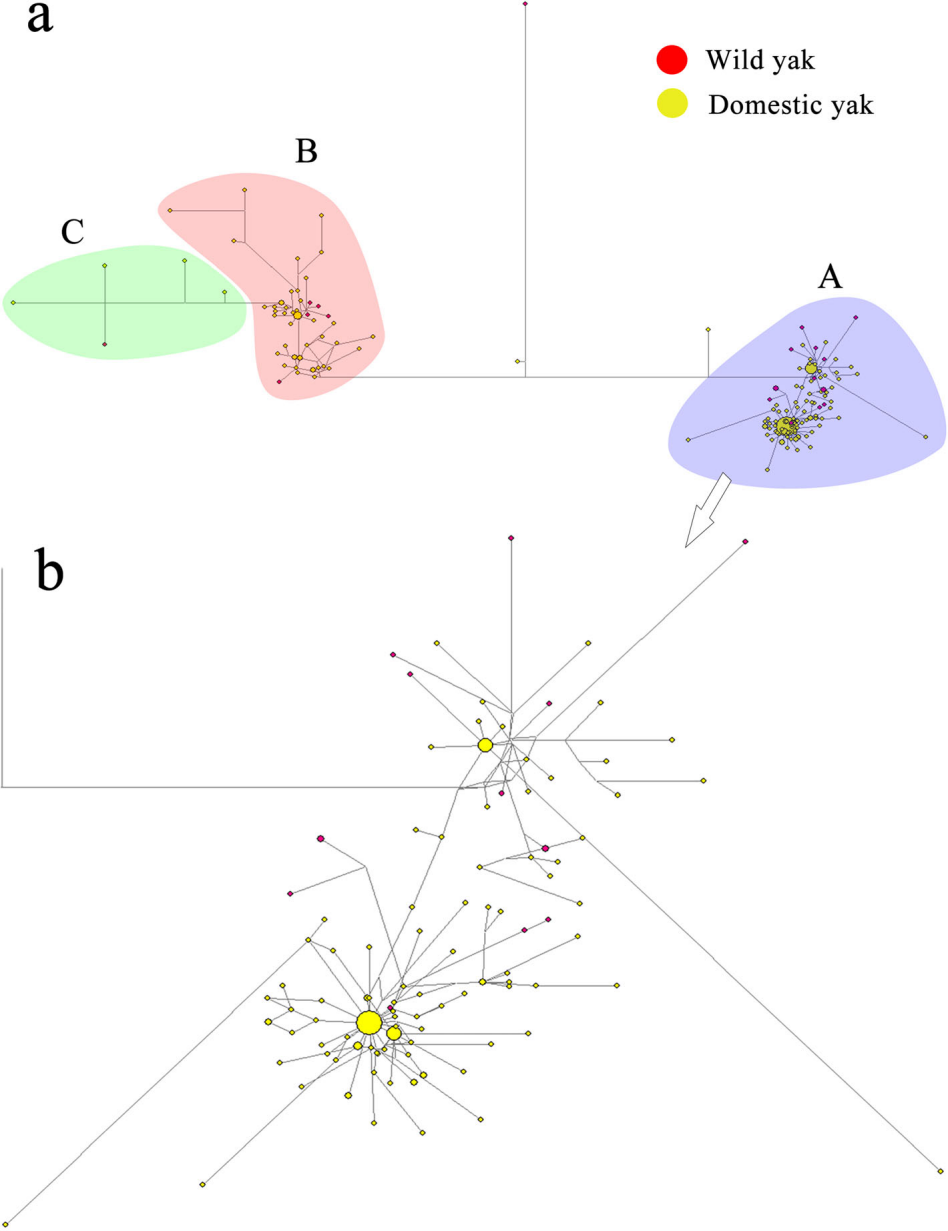
Total network of mitochondrial DNA of 206 individuals. The red dot in the figure represents wild yak, and the yellow dot represents domestic yak. A, B, and C represent three haplogroups. b is an expanded version of haplogroup A.

A network diagram of 206 yak individuals (Fig. 3) was constructed to study the yak phylogeny. This diagram comprised three branches, verifying the reliability of the branches in Fig. 2. Three haplogroups (A–C) were identified, with haplogroups A and B exhibiting a star-shaped phylogenetic relationship and haplogroup C exhibiting a dendritic phylogenetic relationship.

### Analysis of genetic distance between yak populations

Based on the complete mitochondrial DNA sequence, the genetic distances between Yushu yak, Qilian yak, Xueduo yak, Huanhu yak, and Pamir yak populations were calculated. The genetic distance between any two of the five populations was relatively small. The genetic distances between Pamir yak and Qilian yak, and Qilian yak and Xueduo yak were both 0.002, and the genetic distances between other yak groups were all 0.003. The genetic distances among three branches of yaks were calculated based on the complete mitochondrial DNA sequence. The genetic distance between branches I and II was 0.006, between branches I and III was 0.006, and between branches II and III was 0.005. The genetic distance between branches was greater than that between yak populations. Finally, the genetic distance between each haplogroup of yaks was analyzed. They found that the genetic distances between the seven groups, including three haplogroups and four mitotypes, ranged from 0.001 to 0.008. The genetic distance between GQ464260.1 and haplogroup A was the largest, and that between haplogroup A and GQ464276.1 was the smallest. No zero genetic distance existed between any two populations, indicating that the genetic and breeding development of yaks were different. Additional details are shown in Table 2.

**Table 2:** Complete mitochondrial sequence genetic distance between haplogroups and mitotypes

Mitotype/Haplogroup	A	B	C	GQ464260.1	GQ464276.1	x15
B	0.006					
C	0.005	0.002				
GQ464260.1	0.008	0.007	0.008			
GQ464276.1	0.001	0.005	0.005	0.007		
x15	0.004	0.003	0.004	0.004	0.003	
y5	0.002	0.004	0.006	0.007	0.002	0.003

## Discussion

As a form of matrilineally inherited genetic material, mtDNA has been widely used to study maternal phylogenetic relationships among mammals [19, 20]. Studies of the evolution and taxonomy of yak populations in China to date have primarily focused on the mitochondrial D-loop region [21] and cytochrome b (*cytb*) gene sequences [16]. The *cytb* gene is relatively stable in organisms, and its mutation process is slow in mtDNA. Therefore, it is commonly used to reconstruct phylogenetic relationships above the species level. The D-loop region is often used to study the system relationships at the subspecies level because of its rapid variation in mtDNA [15]. Complete mtDNA sequences yield more data than either of these individual isolated sequences, making them more informative than *cytb* gene, D-loop regions, or nuclear genes when evaluating mammalian phylogenetic relationships [22–26]. The four yak populations from Qinghai province and the one yak population from Xinjiang province were sequenced. Their genetic diversity were evaluated by comparing them with one another and with yak mtDNA sequences in the GenBank database to construct a phylogenetic tree of yaks.

Of the 111 individuals, 78 mitotypes were found, among which Yushu yak, Huanhu yak, Xueduo yak, and Qilian yak all shared some mitotypes each other, but the Pamir share no mitotype with these 4 populations. This might be because the first four populations were geographically close to each other and exchanged genes between their populations, while the Pamir yak was geographically far away from the other four populations, creating geographical isolation. Further, the mitotype specificity of the Pamir yak was caused by the accumulation of mutations during migration from its origin to the Pamir region.

Genetic diversity is an integral part of all biological diversity. It is the basis of biological evolution and species differentiation, and is of great significant for population maintenance and reproduction and adaptation to habitat changes. The mitotype diversity and nucleotide diversity are important indicators to measure the degree of genetic variation of the population. The higher the mitotype diversity and nucleotide diversity, the higher the degree of genetic variation of the population. The more the genetic diversity, the more likely it is to adapt to different environments. The mitotype diversity of the five populations was found higher than that of Jiulong yaks, Maiwa yaks, Zhongdian yaks, Tianzhu white yaks, and Huanhu yaks studied by Zhengchao Tu by sequencing the mitochondrial genomes of 111 yaks from 5 populations from Qinghai and Xinjiang [27]. This was consistent with the result that the diversity level of the yak population was the highest in the QTP [14]. The mitotype diversity (0.905 ± 0.048) of Huanhu yaks in this study was close to that of Huanhu yaks (0.9000) studied by Zhengchao Tu, which verified the reliability of the analysis.

Phylogenetic relationship analyses conducted in this study revealed that yak populations were separable into three distinct branches. Compared with the findings of Lai [16], Guo [28], and Ho [29], this experiment identified a new phylogenetic branch with both wild and domestic yaks. However, Wang [30] separated wild yaks into three branches, whereas domestic yaks into two branches. Only a single wild yak was represented in branch III in the present study. In their COIII study of 111 Tibetan yaks, Zhao et al. [31] separated these animals into three branches, consistent with the results of the present study. Highly differentiated, low-frequency mitotypes might be derived from pseudogenes in the nuclear genome that were similar to mitochondrial sequences. However, Wang found that branch III formed by wild yak (GQ464260.1) contained two individuals on the phylogenetic tree in the D-loop region. Meanwhile, a comparison of the nucleotide sequences of mitochondrial protein-coding genes showed that the mitochondrial sequence of the wild yaks forming branch III was very similar to that of other yaks, thus eliminating the possibility of pseudogenes. In this experiment, the mitochondrial sequence clustering analysis revealed that a domestic yak X15 with wild yak (GQ464260.1) formed branch III; through the mitochondrial sequence alignment, X15 and GQ464260.1 had high similarity with other yak mitochondrial sequences. Moreover, the similarity of mitochondrial sequences between X15 and other yaks was higher than that between GQ464260.1 and other yaks. In addition, the evolutionary tree showed that the three highly differentiated genetic branches all had high support rate. Therefore, the experiment excluded the influence of nuclear genes, which verified the reliability of the branch from the side.

Previous studies showed highly differentiated intraspecific genetic branches either from several independent domestication events or from a wild species that diverged in an early stage [32, 33]. All three branches were found in both yaks and wild yaks, suggesting that highly differentiated genetic branches had been developed in the early wild yaks. Differences in grouping among studies might be attributable to limited sample sizes in certain analyses, resulting in the overlooking of yaks in the smaller third phylogenetic branch.

Using mitotype clustering, the 155 mitotypes were divided into 3 haplogroups, while Wang [30] divided yaks into 6 haplogroups based on the mitochondrial coding sequence. Tu [27] used the enzyme digestion method to divide yaks into five haplogroups, revealing that the results of different methods might be inconsistent. Both haplogroups A and B included Yushu yak, Xueduo yak, Huanhu yak, Qilian yak, and Pamir yak. Haplogroup A included yaks from six provinces where yaks were distributed. In addition, the genetic distance analysis showed that the genetic distance between different populations was smaller than that between different branches and between different haplogroups; the genetic distance between different populations was similar. The aforementioned results indicated that the haplogroup was not related to the geographical distribution of yaks, and the cattle from the same population or the same ecological environment were distributed in different haplogroups. Accordingly, each haplogroup contained individuals from different populations or different ecological environments. These results were consistent with the findings of Guo [28] and Wang [30]. In haplogroup B, yak mitochondria from Yunnan province and Tibet were missing. Yak mitochondria were collected from only two yaks from Yunnan province and only four yaks from Tibet. In addition, the sample size of other provinces outside Qinghai province was too small to truly reflect the distribution of their haplogroups. Yaks in Qinghai were also close to the wild yak distribution range, suggesting that Qinghai was likely the site of initial yak domestication [30]. All yaks were divided into three haplogroups; two of these haplogroups (A and B) showed a star-shaped distribution of mitotypes, which was typical of domestic species and consistent with population expansion [34]. The central mitotypes of A and B were widely distributed and had a high frequency. However, the results of this study might be susceptible to haplogroup bias owing to the limited sample size. Additional samples from outside of Qinghai province should be collected to clarify these results in the future.

## Conclusions

In summary, the results revealed that the genetic diversity of yaks in Qinghai was high. Both domestic and wild yaks clustered into three branches.

## Methods

### Animals and sample collection

 The yaks from Qinghai province, the central producing area of yaks, were selected as the research object, and Pamir yaks from Xinjiang were selected as the comparison group. Blood was collected from yaks not related to each other. The yaks had no sex limitation and were aged between 3 and 8 years. A total of 86 yaks from Qinghai province, including 20 yaks from Yushu (N34°7′3″, E95°48′18″), 22 yaks from Qilian (N38°11′42″, E100°16′45″), 23 yaks from Xueduo (N34°44′30″, E101°37′4″), and 21 yaks from Huanhu (N36°55′8″, E98°31′19″), were selected. Further, 25 Pamir yaks from Xinjiang province (N38°22′7″, E75°47′53″) were examined. Venous blood samples were collected from all 111 yaks for mtDNA extraction. The collected blood samples were immediately stored in the in-car refrigerator and transported to the laboratory within 24 h. Then, the blood samples were stored at − 80 °C in a refrigerator of the Yak Breeding Engineering Laboratory of Gansu province. The storage number of blood samples was R-5-1-001, and the DNA was extracted within a month later.

 All procedures involving animals were performed according to the guidelines of the China Council on Animal Care and the Ministry of Agriculture of the People’s Republic of China. The Animal Care and Use Committee of the Lanzhou Institute of Husbandry and Pharmaceutical Sciences Chinese Academy of Agricultural Sciences approved all yak handling procedures (Permit No: SYXK-2014-0002). All blood samples were collected from living yaks, and no yak was sacrificed in this experiment. Before collecting blood samples, the jugular vein of yaks was locally disinfected with alcohol. The blood samples were collected and disinfected with iodine volt to prevent wound infection caused by a needle.

### Extraction, amplification, and sequencing of the mitochondrial genome

 In this study, primers designed by Wang [30] were used to amplify the entire yak mitochondrial genome, and the primers were sent to Xi’an Qingke Biotechnology Co., Ltd. (Xi’an, China) for synthesis. An EasyPure Blood Genomic DNA Kit (Quanshijin Biotechnology Co., Ltd., Beijing, China) was used to extract DNA from yak blood samples. The concentration and OD260/280 values of the extracted DNA were measured using a Nanodrop 2000 spectrophotometer (Thermofisher Scientific, MA, USA). DNA samples with concentrations of 50–1000 ng /µL and OD260/280 values in the range of 1.6–1.8 were selected, and 1 % agarose gel electrophoresis was used to test its integrity. Finally, qualified DNA samples with only one clear band detected by agarose gel electrophoresis were diluted to 50 ng/µL and stored in an ultra-low-temperature refrigerator at − 80 °C for further experiments.

Polymerase chain reaction (PCR) was conducted using a PCR reaction system, with each reaction containing 2 µL of each primer, 25 µL of 2× ProTaq Master Mix, 2 µL of sample DNA, and 19 µL of ddH_2_O. The thermocycler settings were as follows: 95 °C for 3 min; 35 cycles of 95 °C for 20 s, 52 °C for 40 s, and 72 °C for 4 min, followed by 72 °C for 10 min. PCR products were detected by 1 % agarose gel electrophoresis. PCR products with appropriate band size were then sent to Xi’an Qinco Biotechnology Co., Ltd (Xi’an, China) for sequencing.

### Data analysis

MAFFT 7.0 was used for sequence alignment [35], while DNAsp 6.0 [36] was used to calculate the number of variable sites (S), haplotype diversity (Hd), nucleotide diversity (Pi), average number of nucleotide differences (K), and number of haplotypes (H). DAMBE 7 [37] was used to calculate the saturation of sequence base substitutions. MEGA 6 [38] was used to calculate the frontal genetic distance between five yak populations. A neighbor-joining (NJ) tree was constructed, and the reliability of the tree topology was assessed by 1000 bootstrap replications. A median-joining network was constructed using NETWORK 10 [39]. The sequences were deposited in GenBank under the accession numbers MW414100–MW414210.

## Supplementary Information

**Additional file 1: Table S1.** Mitochondrial DNA Haplotype of Qinghai yak and Pamir yak. **Table S2.** Mitochondrial DNA sequence downloaded from GenBank. **Table S3.** The haplotype of 206 mitochondrial DNA. **Table S4.** The accession numbers for mitochondrial DNA.

## Data Availability

The datasets generated and analyzed during the present study are available in the GenBank repository, under the accession number: MW414100–MW414210 (https://www.ncbi.nlm.nih.gov/genbank/). The data supporting the conclusions of this study areavailable in the supplementary table.

## Abbreviations

*Cytb*: Cytochrome b gene
mtDNA: Mitochondrial DNA
NJ: Neighbor-joining
QTP: Qinghai–Tibetan Plateau
