# Supplementary material for: Mitogenomic diversity and phylogeny analysis of yak (Bos grunniens)
Source: BMC Genomics. 2021 May 5;22:325. doi: 10.1186/s12864-021-07650-x (PMC8097944; doi:10.1186/s12864-021-07650-x)
Supplement: Supplementary file 1 — Additional file 1: Table S1. Mitochondrial DNA Haplotype of Qinghai yak and Pamir yak. Table S2. Mitochondrial DNA sequence downloaded from GenBank. Table S3. The haplotype of 206 mitochondrial DNA. Table S4. The accession numbers for mitochondrial DNA. [file 12864_2021_7650_MOESM1_ESM.pdf]

Mitogenomic Diversity and Phylogeny Analysis of Yak (*Bos grunniens*)

Xingdong Wang[1,2], Jie Pei[1], Pengjia Bao[1], Mengli Cao[1], Shaoke Guo[1], Rende Song[3], Weiru Song[3], Chunnian Liang[1], Ping Yan[1]*, and Xian Guo[1]*

[1]Key Laboratory of Yak Breeding Engineering of Gansu Province, Lanzhou Institute of Husbandry and Pharmaceutical Sciences Chinese Academy of Agricultural Sciences, Lanzhou 730050, China.

[2]Life Science and Engineering College, Northwest University for Nationalities, Lanzhou 730030, China.

[3]Animal Disease Prevention and Control Center of Yushu Tibetan Autonomous Prefecture, Yushu 815000, China.

*Corresponding authors: Xian Guo (email: guoxian@caas.cn) and Ping Yan (email: pingyanlz@163.com)

Table S1. Mitochondrial DNA Haplotype of Qinghai yak and Pamir yak

Mitotype	Number	Sequence name
H1	5	h1; h13; q1; q12; x13
H2	13	h2; h8; h11; h12; h14; h19; q3; q11; q14; q18; q21; x11; y11
H3	1	h3
H4	2	h4; q17
H5	1	h5
H6	1	h6
H7	1	h7
H8	2	h9; h17
H9	2	h10; y5
H10	4	h15; h20; h21; q4
H11	1	h16
H12	1	h18
H13	1	p1
H14	1	p2
H15	1	p3
H16	1	p4
H17	1	p5
H18	1	p6
H19	1	p7
H20	1	p8
H21	1	p9
H22	1	p10
H23	2	p11; p12
H24	1	p13
H25	1	p14
H26	1	p15
H27	2	p16; p19
H28	2	p17; p21
H29	1	p18
H30	1	p20
H31	1	p22
H32	1	p23
H33	1	p24
H34	1	p25
H35	1	q2
H36	1	q5
H37	1	q6
H38	1	q7
H39	1	q8
H40	1	q9
H41	2	q10; q13
H42	1	q15

Mitotype	Number	Sequence name
H43	1	q16
H44	1	q19
H45	1	q20
H46	1	q22
H47	6	x1; x5; y1; y4; y6; y8
H48	2	x2; x9
H49	1	x3
H50	1	x4
H51	1	x6
H52	1	x7
H53	1	x8
H54	1	x10
H55	1	x12
H56	1	x14
H57	1	x15
H58	1	x16
H59	1	x17
H60	1	x18
H61	1	x19
H62	1	x20
H63	1	x21
H64	1	x22
H65	1	x23
H66	1	y2
H67	1	y3
H68	1	y5
H69	1	y7
H70	1	y9
H71	1	y10
H72	1	y12
H73	1	y13
H74	1	y14
H75	1	y16
H76	1	y17
H77	2	y18; y20
H78	1	y19

Note: h represents the Huanhu yak, y represents the Yushu yak, x represents the Xueduo yak, q represents the Qilian yak, and p represents the Pamir yak.

Table S2. Mitochondrial DNA sequence downloaded from GenBank

Province	Breed	Number	GenBank accession number
Xinjiang	Bazhou	1	MN175233.1
Yunnan	Zhongdian	2	GQ464314.1; MT850134.1

Province	Breed	Number	GenBank accession number
Tibet	Pali	1	KR052524.1
	Sibu	1	MN398192.1
	Niangya	1	MN319467.1
Gansu	Sunan	1	MH921427.1
	Gannan	8	GQ464267.1 ~ GQ464273.1; KJ704989.1
	Tianzhu	4	GQ464274.1 ~ GQ464277.1
Sichuan	Jinchuan	1	MN176980.1
	Maiwa	12	KX232521.1 ~ KX232527.1; GQ464300.1 ~ GQ464302.1; GQ464304.1; GQ464306.1
	Jiulong	2	GQ464299.1; GQ464305.1
	Hongyuan	1	MT162465.1
Qinghai	Tianjun	1	MN163006.1
	Datong	3	KJ463418.1; GQ464289.1 ~ GQ464290.1
	Huanhu	5	GQ464285.1 ~ GQ464288.1; GQ464291.1
	Yushu	7	GQ464292.1 ~ GQ464298.1
	Ashdan	1	KM658599.1
	Wild yak	21	GQ464246.1 ~ GQ464266.1
Unclear	Unclear	22	GQ464278.1 ~ GQ464284.1; GQ464307.1 ~ GQ464313.1; JQ846021.1 ~ JQ846022.1; EF494177.1 ~ EF494179.1; JQ437480.1; MF973066.1; MT649465.1

Table S3. The haplotype of 206 mitochondrial DNA

Mitotype	Number	Sequence name
H1	8	h1, h13, q1, q12, x13, GQ464312.1, GQ464281.1, GQ464301.1
H2	22	h2, h8, h11, h12, h14, h19, q3, q11, q14, q18, q21, x11, y11, GQ464308.1, MN176980.1, GQ464271.1, GQ464273.1, GQ464274.1, GQ464277.1, GQ464285.1, MT850134.1, MN398192.1
H3	1	h3
H4	2	h4, q17
H5	1	h5
H6	1	h6
H7	1	h7
H8	2	h9, h17
H9	3	h10, y15, GQ464289.1

Mitotype	Number	Sequence name
H10	4	h15, h20, h21, q4
H11	1	h16
H12	1	h18
H13	1	p1
H14	1	p2
H15	1	p3
H16	1	p4
H17	1	p5
H18	1	p6
H19	1	p7
H20	1	p8
H21	1	p9
H22	1	p10
H23	2	p11, p12
H24	1	p13
H25	1	p14
H26	1	p15
H27	2	p16, p19
H28	2	p17, p21
H29	1	p18
H30	1	p20
H31	1	p22
H32	1	p23
H33	1	p24
H34	1	p25
H35	1	q2
H36	1	q5
H37	1	q6
H38	1	q7
H39	1	q8
H40	1	q9
H41	2	q10, q13
H42	1	q15
H43	1	q16
H44	1	q19
H45	1	q20
H46	1	q22
H47	7	x1, x5, y1, y4, y6, y8, MT649465.1
H48	2	x2, x9
H49	1	x3
H50	1	x4
H51	1	x6
H52	1	x7

Mitotype	Number	Sequence name
H53	1	x8
H54	1	x10
H55	1	x12
H56	1	x14
H57	1	x15
H58	1	x16
H59	1	x17
H60	1	x18
H61	1	x19
H62	1	x20
H63	1	x21
H64	1	x22
H65	1	x23
H66	1	y2
H67	1	y3
H68	1	y5
H69	1	y7
H70	1	y9
H71	1	y10
H72	1	y12
H73	1	y13
H74	1	y14
H75	1	y16
H76	1	y17
H77	2	y18, y20
H78	1	y19
H79	1	GQ464311.1
H80	1	GQ464313.1
H81	1	GQ464310.1
H82	1	GQ464309.1
H83	1	GQ464307.1
H84	1	GQ464284.1
H85	1	GQ464283.1
H86	1	GQ464282.1
H87	1	GQ464280.1
H88	1	GQ464279.1
H89	2	GQ464278.1, GQ464269.1
H90	1	JQ846022.1
H91	1	JQ846021.1
H92	1	JQ437480.1
H93	1	MF973066.1
H94	1	EF494179.1
H95	1	EF494178.1

Mitotype	Number	Sequence name
H96	1	EF494177.1
H97	1	MN163006.1
H98	1	KR052524.1
H99	1	KX232521.1
H100	1	KX232522.1
H101	1	KX232523.1
H102	1	KX232524.1
H103	1	KX232525.1
H104	1	KX232526.1
H105	1	KX232527.1
H106	1	KJ463418.1
H107	1	MH921427.1
H108	1	GQ464267.1
H109	1	GQ464268.1
H110	1	GQ464270.1
H111	1	GQ464272.1
H112	1	GQ464275.1
H113	1	GQ464276.1
H114	1	GQ464286.1
H115	1	GQ464287.1
H116	1	GQ464288.1
H117	1	GQ464290.1
H118	2	GQ464291.1, GQ464296.1
H119	1	GQ464292.1
H120	1	GQ464293.1
H121	1	GQ464294.1
H122	1	GQ464295.1
H123	1	GQ464297.1
H124	1	GQ464298.1
H125	1	GQ464299.1
H126	1	GQ464305.1
H127	1	GQ464306.1
H128	1	GQ464300.1
H129	1	GQ464302.1
H130	1	GQ464304.1
H131	1	GQ464314.1
H132	1	MN319467.1
H133	1	MN175233.1
H134	1	KM658599.1
H135	1	KJ704989.1
H136	1	MT162465.1
H137	2	GQ464246.1, GQ464264.1
H138	1	GQ464247.1

Mitotype	Number	Sequence name
H139	1	GQ464248.1
H140	1	GQ464249.1
H141	1	GQ464250.1
H142	1	GQ464251.1
H143	2	GQ464252.1, GQ464253.1
H144	1	GQ464254.1
H145	1	GQ464255.1
H146	1	GQ464256.1
H147	1	GQ464257.1
H148	1	GQ464258.1
H149	1	GQ464259.1
H150	1	GQ464260.1
H151	1	GQ464261.1
H152	1	GQ464262.1
H153	1	GQ464263.1
H154	1	GQ464265.1
H155	1	GQ464266.1

Table S4. The accession numbers for mitochondrial DNA

**Blood sample**	**Accession number**
h1	MW414100
h2	MW414101
h3	MW414102
h4	MW414103
h5	MW414104
h6	MW414105
h7	MW414106
h8	MW414107
h9	MW414108
h10	MW414109
h11	MW414110
h12	MW414111
h13	MW414112

Blood sample	Accession number
h14	MW414113
h15	MW414114
h16	MW414115
h17	MW414116
h18	MW414117
h19	MW414118
h20	MW414119
h21	MW414120
q1	MW414121
q2	MW414122
q3	MW414123
q4	MW414124
q5	MW414125
q6	MW414126
q7	MW414127
q8	MW414128
q9	MW414129
q10	MW414130
q11	MW414131
q12	MW414132
q13	MW414133
q14	MW414134
q15	MW414135
q16	MW414136
q17	MW414137

Blood sample	Accession number
q18	MW414138
q19	MW414139
q20	MW414140
q21	MW414141
q22	MW414142
x1	MW414143
x2	MW414144
x3	MW414145
x4	MW414146
x5	MW414147
x6	MW414148
x7	MW414149
x8	MW414150
x9	MW414151
x10	MW414152
x11	MW414153
x12	MW414154
x13	MW414155
x14	MW414156
x15	MW414157
x16	MW414158
x17	MW414159
x18	MW414160
x19	MW414161
x20	MW414162

Blood sample	Accession number
x21	MW414163
x22	MW414164
x23	MW414165
p1	MW414166
p2	MW414167
p3	MW414168
p4	MW414169
p5	MW414170
p6	MW414171
p7	MW414172
p8	MW414173
p9	MW414174
p10	MW414175
p11	MW414176
p12	MW414177
p13	MW414178
p14	MW414179
p15	MW414180
p16	MW414181
p17	MW414182
p18	MW414182
p19	MW414184
p20	MW414185
p21	MW414186
p22	MW414187

Blood sample	Accession number
p23	MW414188
p24	MW414189
p25	MW414190
y1	MW414191
y2	MW414192
y3	MW414193
y4	MW414194
y5	MW414195
y6	MW414196
y7	MW414197
y8	MW414198
y9	MW414199
y10	MW414200
y11	MW414201
y12	MW414202
y13	MW414203
y14	MW414204
y15	MW414205
y16	MW414206
y17	MW414207
y18	MW414208
y19	MW414209
y20	MW414210
